# SVM-SulfoSite: A support vector machine based predictor for sulfenylation sites

**DOI:** 10.1038/s41598-018-29126-x

**Published:** 2018-07-26

**Authors:** Hussam J. AL-barakati, Evan W. McConnell, Leslie M. Hicks, Leslie B. Poole, Robert H. Newman, Dukka B. KC

**Affiliations:** 1 Department of Computational Science and Engineering, North Carolina A&T State University, Greensboro, NC 27411 USA; 2 Department of Chemistry, University of North Carolina at Chapel Hill, Chapel Hill, NC 27599 USA; 3 Department of Biochemistry, Wake Forest University School of Medicine, Winston-Salem, NC 27157 USA; 4 Department of Biology, North Carolina A&T State University, Greensboro, NC 27411 USA

## Abstract

Protein *S*-sulfenylation, which results from oxidation of free thiols on cysteine residues, has recently emerged as an important post-translational modification that regulates the structure and function of proteins involved in a variety of physiological and pathological processes. By altering the size and physiochemical properties of modified cysteine residues, sulfenylation can impact the cellular function of proteins in several different ways. Thus, the ability to rapidly and accurately identify putative sulfenylation sites in proteins will provide important insights into redox-dependent regulation of protein function in a variety of cellular contexts. Though bottom-up proteomic approaches, such as tandem mass spectrometry (MS/MS), provide a wealth of information about global changes in the sulfenylation state of proteins, MS/MS-based experiments are often labor-intensive, costly and technically challenging. Therefore, to complement existing proteomic approaches, researchers have developed a series of computational tools to identify putative sulfenylation sites on proteins. However, existing methods often suffer from low accuracy, specificity, and/or sensitivity. In this study, we developed SVM-SulfoSite, a novel sulfenylation prediction tool that uses support vector machines (SVM) to identify key determinants of sulfenylation among five feature classes: binary code, physiochemical properties, k-space amino acid pairs, amino acid composition and high-quality physiochemical indices. Using 10-fold cross-validation, SVM-SulfoSite achieved 95% sensitivity and 83% specificity, with an overall accuracy of 89% and Matthew’s correlation coefficient (MCC) of 0.79. Likewise, using an independent test set of experimentally identified sulfenylation sites, our method achieved scores of 74%, 62%, 80% and 0.42 for accuracy, sensitivity, specificity and MCC, with an area under the receiver operator characteristic (ROC) curve of 0.81. Moreover, in side-by-side comparisons, SVM-SulfoSite performed as well as or better than existing sulfenylation prediction tools. Together, these results suggest that our method represents a robust and complementary technique for advanced exploration of protein S-sulfenylation.

## Introduction

**Figure 1:**
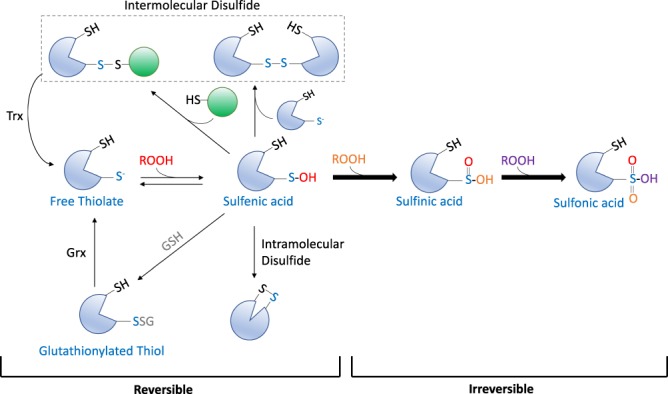
In the presence of an oxidizing agent (ROOH), the thiolate anion of a redox-sensitive Cys (Cys-S^−^) is reversibly oxidized to form a sulfenic acid (SOH). When in close proximity to another reactive Cys (SH), either in the same protein molecule or in another protein, SOH leads to disulfide bond (S-S) formation. In addition to Cys residues in proteins, SOH can also react with the cellular antioxidant, glutathione (g-Glu-Cys-Gly; GSH) to form a mixed S-S bond (PSSG). Aside from altering the chemical properties of the Cys residue and the tertiary structure of the protein, S-S bonds are also believed to prevent terminal oxidation to sulfinic (SO_2_H) and sulfonic (SO_3_H) acid. Disulfide bonds can be reduced back to the thiol by cellular antioxidant enzymes, such as glutaredoxin (Grx) or thioredoxin (Trx).

Redox-dependent signalling plays a critical role in physiological processes such as aging and the immune response, as well as in a number of pervasive diseases, including cancer, Alzheimer’s disease, cardiovascular disease and diabetes^1–3^. For instance, protein S-sulfenylation (Fig. 1), which typically occurs in the cell via H_2_O_2_–dependent conversion of free thiols (-SH) on cysteine residues to sulfenic acid (-SOH), has emerged as a key post-translation modification (PTM) that can regulate the function of target proteins^4,5^. For example, sulfenylation can modulate the enzymatic activity, binding affinity, stability and/or subcellular localization of cellular proteins^2,5–8^. While sulfenylation is readily reversible (either by reduction back to the free thiol or via disulfide formation followed by reduction by cellular enzymes, such as glutaredoxin and thioredoxin), further oxidation by various peroxides (ROOH) or H_2_O_2_ can convert sulfenic acid to sulfinic and sulfonic acid moieties that are largely irreversible inside the cell^9^.

To identify sulfenylation sites on proteins, several chemo-proteomic methods have been developed^10–16^. For instance, Yang *et al*. recently used selective labelling for cysteine sulfenic acid together with tandem mass spectrometry (MS/MS)-based detection to identify more than 1,000 sulfenylation sites in ~700 cellular proteins^4^.Though such chemo-proteomic methods provide a wealth of information about the redox regulation of cellular proteins *in situ*, these methods are time-consuming, expensive and require a high level of technical expertise. Moreover, though MS-based methods provide significant depth of coverage of the ‘sulfenylome’, they can be biased towards abundant proteins. Finally, other redox reactions that occur following lysis, such as adventitious oxidation by ambient oxygen, can generate SOH sites that do not necessarily occur under physiological conditions. Therefore, to complement global proteomic analysis of sulfenylation site identification, several computational approaches have been developed to predict sulfenylation sites *in silico*^17–22^. The first method, developed by Bui and colleagues in 2015, uses maximal dependence decomposition (MDD) and support vector machines (SVM) to identify sulfenylation motifs based on amino acid composition and solvent accessibility. Using 5-fold cross-validation, the resulting method, known as MDD-SOH, achieved efficiency scores of 68%, 70%, 70%, and 0.27 for accuracy (ACC), sensitivity (SN), specificity (SP), and Matthew’s correlation coefficient (MCC), respectively^17^. Subsequently, Xu *et al*. used SVM to identify key parameters for sulfenylation from among 14 types of physicochemical properties^18^. The resulting sulfenylation site prediction tool, termed iSulf-Cys, performed similarly to MDD-SOH based on 10-fold cross-validation, with a slight increase in MCC^18^. Similarly, other prediction tools based on strategies including position specific scoring matrices (PSSM) and SVM have offered modest improvements in performance^19–21^. For instance, Sakka *et al*. developed the PRotEin S-sulfenylation server (PRESS), which uses SVM to identify structural features that correlate with sulfenylation^20^. Using 10-fold cross-validation, PRESS achieved ACC, SN and SP scores of 77%, 80% and 74%, respectively. Most recently, two prediction tools, S-SulfPred and SulCysSite, were reported that also show significant improvements in one or more performance metric^22,23^. For instance, Hasan *et al*. developed a predictor named SulCysSite that uses a random forest-based strategy to identify key parameters related to sulfenylation from among four features. Using this approach, the authors observed SN, SP and MCC scores of 62%, 81%, and 0.45, respectively, upon 10-fold cross-validation^23^. Likewise, Jia and Zuo developed a method named S-SulfPred, which combines a synthetic minority oversampling technique (SMOTE) and an under-sampling strategy with an SVM implementation to predict sulfenylation sites based on three feature types^22^. Upon 10-fold cross-validation, S-SulfPred achieved impressive scores of 88%, 78%, 91% and 0.64 in terms of ACC, SN, SP and MCC, respectively.

Despite the significant progress that has been made over the past two years, there is still room for improvement in the performance of existing sulfenylation site prediction tools. For instance, many of the existing sulfenylation site prediction tools exhibit relatively low MCC scores compared to prediction tools for other posttranslational modifications, such as phosphorylation^24^, hydroxylation^25^ and glycosylation^26^. While this is likely a function of the size of datasets available for training and/or the mode of modification (*i.e*., enzyme-mediated modification for phosphorylation, hydroxylation and glycosylation versus predominantly non-enzymatic modification for sulfenylation), it is also likely that other factors contribute to the relatively low MCC scores. Moreover, in addition to the primary amino acid sequence, some methods, such as PRESS, require structural data about the protein-of-interest. As a consequence, the number of proteins that can be analysed is greatly reduced. Here, we describe the development and evaluation of SVM-SulfoSite, a novel sulfenylation site prediction tool designed to identify putative sulfenylation sites using only the primary amino acid sequence as input. SVM-SulfoSite combines multiple features, including physiochemical properties, amino acid composition and high-quality indices, with novel classifier algorithms and an SVM-based machine learning strategy to predict sulfenylation sites in proteins. Based on evaluation using both 10-fold cross-validation and an independent dataset, SVM-SulfoSite compares favourably to existing sulfenylation site prediction tools with regard to accuracy, sensitivity, specificity and MCC. Therefore, SVM-SulfoSite represents a robust, accessible sulfenylation prediction tool that promises to provide additional insights into the regulation and biological consequences of protein *S*-sulfenylation.

## Results and Discussion

### Model development

To develop a robust sulfenylation site prediction tool that is able to identify putative sulfenylation sites using only the primary amino acid sequence as input, we first compiled training and independent test sets similar to those described by Xu *et al*.^18^. After discarding sequences that exhibited ≥40% identity, 1,045 positive sites and 7,126 negative sites on 778 human proteins remained. All sites had been experimentally verified previously^4^. We then randomly selected 145 positive sites and 268 negative sites for the independent test set and used the remaining sites for model training and development. The training set consisted of 900 positive sites and 6,858 negative sites. Each 21-residue fragment contained a central cysteine (either sulfenylated or not sulfenylated) flanked by 10 residues on either side. Due to the disproportionate number of negative sites, the positive and negative sets were imbalanced. Therefore, we used oversampling to balance the positive and negative classes^27^. This approach, which randomly selects data points from the minority class until it is equivalent to the majority class, has been used for bioinformatics analysis in several biological contexts^28,29^. After balancing the positive and negative training sets, all fragment sequences were converted into vectors for analysis. To this end, we selected five features—binary encoding (BE), 14 types of physicochemical amino acid properties (AAindex), k-spaced amino acid pairs (KSAAP), amino acid composition (AAC) and high-quality indices (HQI)—and generated a unique vector for each feature. Finally, to build our predictor, a SVM-based machine learning strategy was employed to identify those parameters that correlated with sulfenylation in the training set. After each round of parameter optimization, the resulting model was assessed using 10-fold cross-validation until no further improvement in model performance was observed. The resulting model, which we termed SVM-SulfoSite, exhibited robust performance with respect to several standard scoring metrics, as described below.

### Model evaluation

**Figure 2:**
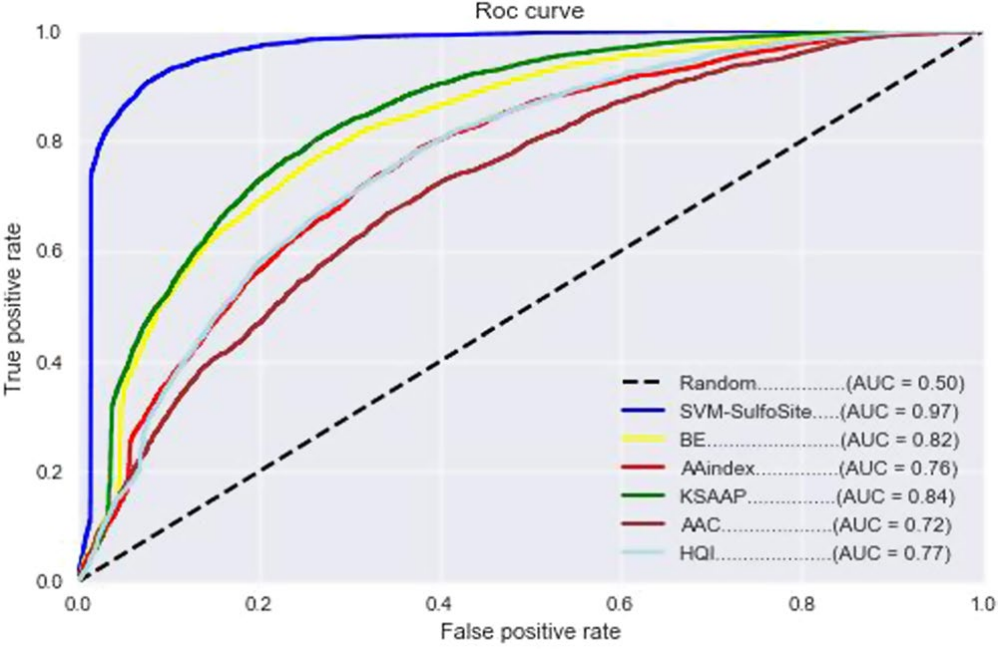
Receiver operator characteristic (ROC) curves for each of five features used to develop our method as well as that for the final method utilizing all features (SVM-SulfoSite) for the 10-fold cross-validation. The area under the curve (AUC) for each feature is given in parentheses.

To assess the performance of SVM-SulfoSite, we first used 10-fold cross-validation^30–33^. To this end, the training set was partitioned into 10 subsets. We then used 9 of the subsets to train the model and the remaining subset for testing. We repeated this technique ten times, with the final result representing the average performance of 10 models. As can be seen in Table 1, SVM-SulfoSite exhibited high efficiency scores for ACC, SN and SP, leading to a strong MCC score of 0.79. Though the scores were relatively high across all metrics, SVM-SulfoSite performed particularly well with respect to SN (95%) and ACC (89%). Likewise, the area under the ROC curve was a robust 0.97 (Fig. 2). To gain further insights into the relative impact of each feature on the overall performance of our method, we also conducted 10-fold cross-validation using only one feature at a time (Table 1 and Fig. 2). While each of the individual features contributed substantially to the overall score for most metrics, KSAAP appears to have the largest impact on model performance across all metrics except SP. Interestingly, while BE and AAC both appear to have a relatively strong effect on sensitivity, AAC does not impact specificity to the same extent as the other features. Together, these data suggest that both the frequency (AAC) and the relative spacing of amino acids around the modified cysteine residue (KSAAP) may play an important role in correctly identifying positive sites of sulfenylation (i.e., SN) while the former is less important for correctly predicting negative sites (i.e., SP).

**Table 1:** Results of 10-fold cross-validation using individual and cumulative features.

Features	Performance (%)			
	ACC	SN	SP	MCC
BE	74	81	69	0.49
AAindex	70	73	66	0.39
KSAAP	76	85	67	0.53
AAC	65	76	55	0.31
HQI	70	73	66	0.39
All Features	89	95	83	0.79

In addition to 10-fold cross-validation, we also evaluated the performance of our method using an independent test set that had been kept hidden from the model during training. Though the efficiency scores were not as high as those observed using 10-fold cross-validation, SVM-SulfoSite still performed quite well using the independent test set (Table 2). Interestingly, while very little difference was observed between the SP scores obtained using the independent set and 10-fold cross-validation (80% and 83%,respectively), we noticed a marked decrease in SN using the independent set (62% for the independent test set versus 95% for 10-fold cross-validation). Likewise, there was a modest decrease in ACC between the two evaluation methods (74% for the independent set and 89% for 10-fold cross-validation). Together, these changes decreased the MCC score from 0.79 to 0.42. Likewise, the AUC dropped from 0.97 to 0.81. The observed differences between the performance metrics using 10-fold cross-validation and our independent set may be a consequence of the oversampling strategy used to balance the positive and negative datasets during training. Among the individual features, KSAAP scores decreased most dramatically when SVM-SulfoSite was evaluated using the independent test set, followed by BE. On the other hand, AAindex and HQI exhibited very similar scores regardless of the evaluation method used. It is interesting to note that both of these features rely on physiochemical properties of amino acids. This is consistent with the notion that sulfenylation is typically a non-enzymatic PTM that may be particularly sensitive to the surrounding physiochemical environment of the modified cysteine^7^.

**Table 2:** Independent test result using individual and cumulative features.

Features	Performance (%)			
	ACC	SN	SP	MCC
BE	68	66	69	0.34
AAindex	65	68	63	0.30
KSAAP	65	72	62	0.32
AAC	61	74	53	0.26
HQI	68	71	66	0.35
All Features	74	62	80	0.42

### Comparison with existing methods

Next, we compared the performance of SVM-SulfoSite with that of other recently developed sulfenylation site predictors using both 10-fold cross-validation and independent test sets. Based on 10-fold cross-validation, SVM-SulfoSite performed as well or better than all existing methods in each category (with the exception of SP, where SVM-SulfoSite was outperformed by S-SulfPred) (Table 3). The largest improvements were observed for MCC, where SVM-SulfoSite scored ~2.1-fold higher than the average MCC across all methods (ranging from a 23% improvement versus S-SulfPred to a 2.9-fold improvement over MDD-SOH). This was followed by SN, where SVM-SulfoSite exhibited an average increase of 36% (ranging from 19 to 61% over PRESS and SOHPRED, respectively) and ACC (average improvement of 19%, ranging from 1 to 35% improvement over S-SulfPred and iSulf-Cys, respectively). Finally, the smallest gains were observed for SP, where SVM-SulfoSite exhibited an average improvement of 11% (ranging from a 9% decrease versus S-SulfPred to a 30% increase compared to iSulf-Cys).

**Table 3:** Comparison of sulfenylation site predictors using 10-fold cross-validation.

Predictor	Performance (%)			
	ACC	SN	SP	MCC
iSulf-Cys	66	67	64	0.31
MDD-SOH	70	68	70	0.27
SOHSite	74	74	74	0.33
SOHPRED	—	59	—	0.28
PRESS	77	80	74	—
SulCysSite	—	62	81	0.45
S-SulPred	88	78	91	0.64
SVM-SulfoSite	89	95	83	0.79

Interestingly, SP was one of the categories where we observed the greatest gains when the methods were compared using an independent test set (Table 4). In fact, the average increase in SP observed for SVM-SulfoSite using the independent dataset (18%) was ~65% greater than that observed using 10-fold cross-validation (11%). The gains in SP ranged from a 13% increase (which was observed for several methods, including S-SulfPred) to a 21% increase over iSulf-Cys. Similarly, SVM-SulfoSite outperformed all existing methods with respect to ACC using the independent set. Indeed, the average improvement in ACC was 6%, ranging from 3 to 10% versus S-SulfPred and iSulf-Cys, respectively. In contrast, SVM-SulfoSite exhibited lower SN scores than the other methods, with an average decrease of 14% (ranging from 10% versus iSulf-Cys to 18% compared to SulCysSite). Nonetheless, the improvements observed in the other areas led to an average increase in MCC of 33% for SVM-SulfoSite when assessed using the independent test set (ranging from a 2% decrease versus S-SulfPred to a 56% increase versus PRESS). Together, these data suggest that SVM-SulfoSite is able to distinguish between sulfenylated and non-sulfenylated cysteine residues in an efficient manner. Importantly, our predictor performs as well as or better than existing methods in all performance metrics except SN, where it exhibits modest decreases in performance when using an independent dataset. In particular, SVM-SulfoSite performs very well with respect to SP, where it outperforms existing methods in side-by-side comparisons using an independent dataset. These data suggest that SVM-SulfoSite is able to distinguish between true negative and false positive sites more efficiently than existing tools.

**Table 4:** Comparison of sulfenylation site predictors using an independent test set.

Predictor	Performance (%)			
	ACC	SN	SP	MCC
iSulf-Cys	64	69	66	0.33
MDD-SOH	71	71	71	0.30
SOHSite	69	72	69	0.28
SOHPRED	—	73	71	0.32
PRESS	—	68	69	0.27
SulCysSite	—	76	71	0.34
S-SulPred	72	75	71	0.43
SVM-SulfoSite	74	62	80	0.42

## Conclusion

Reversible sulfenylation of proteins is an important PTM involved in the regulation of a number cellular processes that contribute to health and disease, yet experimental identification of sulfenylation sites remains challenging. This is due to several factors, including the highly reactive nature of the sulfenic acid moiety and the fact that sulfenylation is generally believed to be a non-enzymatic process^7^. Therefore, several computational methods have recently been developed to predict putative sulfenylation sites *in silico*. Despite these strides, there is still room for improvement - particularly with respect to overall predictor performance. Here, we developed a novel sulfenylation prediction tool, termed SVM-SulfoSite, that uses a SVM-based strategy to identify parameters that correlate with sulfenylation among five distinct feature sets. Based on analysis using both 10-fold cross-validation and an independent dataset, SVM-SulfoSite performed as well or better than existing methods with respect to MCC (Tables 3 and 4), which is often viewed as a surrogate for overall performance since it integrates information about the true positive, false positive, true negative and false negative rates^34,35^. For all previously developed sulfenylation prediction methods, the MCC is <0.65 based on 10-fold cross-validation (Table 3). In contrast, SVM-SulfoSite achieved an MCC of 0.79 when assessed using 10-fold cross-validation. Similarly, when performance was assessed using an independent test set, only S-SulfPred and SVM-SulfoSite achieved an MCC >0.42 (Table 4).

In particular, SVM-SulfoSite consistently ranked among the best methods (i.e., either first or second) with respect to ACC, SP and MCC regardless of the evaluation method employed. Interestingly, though SVM-SulfoSite exhibited the highest SN score when assessed by 10-fold cross-validation (95%), its performance in this area decreased substantially when evaluated using an independent test set (62%). This may be a consequence of the oversampling strategy that we employed to account for incongruities between the number of positive and negative sites in the training set. Indeed, due to the sub-stoichiometric nature of many PTMs inside the cell, a major challenge in PTM predictor development is how to handle imbalanced datasets. Whereas under-sampling is often used to balance training sets during the development of prediction tools for well-studied PTMs such as phosphorylation sites^24,36^, due to the relatively small number of experimentally-verified sulfenylation sites available for analysis, we chose instead to resolve this issue using an oversampling strategy so that no information about negative sites was lost during balancing. On the other hand, this strategy likely improved the performance of SVM-SulfoSite with respect to SP. Indeed, since negative sites were retained during training, our method is able to identify negative sites more efficiently than other methods, as reflected in the highest SP scores using both 10-fold cross-validation and an independent test set. Similarly, the ability to better distinguish between positive and negative sites may have also contributed to the improved fidelity of our method, which is supported by increased scores for ACC and MCC. In conclusion, we have developed and evaluated a novel and robust sulfenylation site prediction tool, termed SVM-SulfoSite, that is able to efficiently identify sulfenylation sites in proteins using only the primary amino acid sequence as input. As a complementary approach to experimental strategies, such as chemo-proteomic and biochemical analyses, SVM-SulfoSite has the potential to provide new insights into the characteristics and functional consequences of protein sulfenylation. To facilitate its use by the biomedical research community, the method has been posted on GitHub (https://github.com/HussamAlbarakati/SVM-Sulfosite) and we are currently developing a webserver that will allow biological researchers to use this method to predict putative sulfenylation sites in proteins-of-interest.

## Materials and Methods

**Figure 3:**
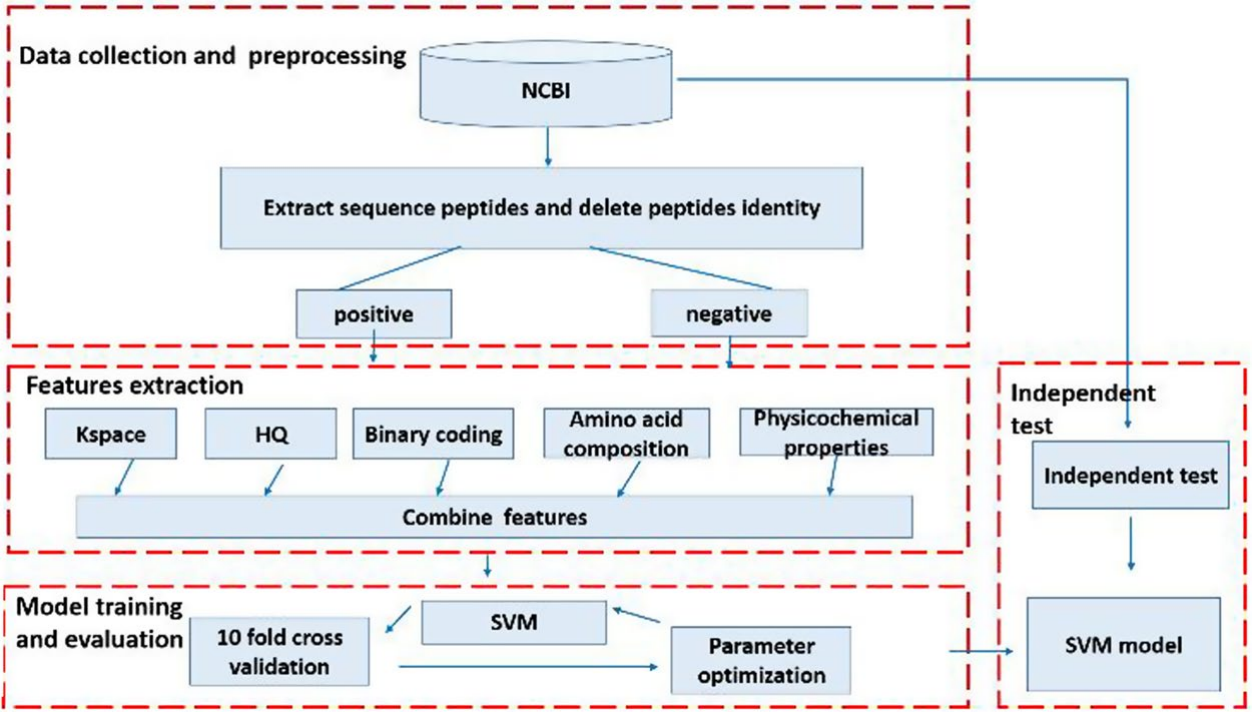
Schematic showing the workflow used to develop our method. KSAAP: k-space amino acid pairs; HQI: high-quality indices; BE: Binary encoding; AAC: Amino acid composition; AAindex: physiochemical amino acid properties; SVM: support vector machines; NCBI: National Center for Biotechnology Information.

Our method was developed using a four-step procedure (Fig. 3). In the first step, data were collected and pre-processed to remove overlapping sequences. Next, sequences containing a central cysteine residue were identified and features (defined below) were extracted using binary encoding (BE), 14 types of physiochemical amino acid properties (AAindex), k-space amino acid pairs (KSAAP), amino acid composition (AAC) and high-quality indices (HQI). Following feature extraction, oversampling techniques were used to balance the training dataset. Finally, the model was evaluated using both 10-fold cross-validation and an independent dataset.

### Binary encoding (BE)

To convert each amino acid in a sequence into a unique numerical vector, we used an orthogonal binary scheme. According to this strategy, 21 dimensional vectors were used to represent each of the 20 canonical amino acid plus a 21^st^ position, corresponding to non-existent residues at the extreme N- or C-terminus of a protein (denoted as X). For example, alanine (A) was encoded as “100000000000000000000”, while cysteine (C) was encoded as “010000000000000000000” and so on. “Non-existent” X residues were encoded as “000000000000000000001”. In all windows evaluated, the central residue was cysteine (C), which was removed from window sites. Therefore, the total BE feature vector was 420.

### Amino acid index (AAindex) property

Each amino acid has several specific physicochemical and biological properties that directly or indirectly influence protein properties. Dissimilar mixtures of those properties have different impacts to the construction and function of proteins. AAindex^37^ is a database that holds numerous physicochemical and biological properties of amino acids. Several mixtures of physicochemical properties have been employed that effectively convert sequences of peptides into mathematical expressions^18,38–40^. In this study, we used 14 physicochemical properties: hydrophobicity, solvent accessibility, polarity, polarizability, accessibility, *PK-N*, *PK-C*, melting point, molecular weight, optical rotation, net charge index of side chains, entropy of formation, heat capacity and absolute entropy. In each case, the feature properties for non-existent residues, X, were represented as 0. Likewise, as above, the central residue was always Cys, which was not considered for window sites. Therefore, the total AAindex feature vector was 280.

### K-space amino acid pairs (KSAAP)

Each KSAAP can be denoted as *f*_*r*_*{k}f*_*s*_(*r*, s = 1, 2, …, 20), where *f*_*r*_ and *f*_*s*_ represent any two residues from among the 20 canonical amino acids. If *k* = 0, *f*_*r*_*{k}f*_*s*_ positions for a dipeptide and total feature vector is 400^41^. In this study, we also considered X, which represents non-existent residues such that *f*_*r*_*{k}f*_*s*_(*r*, s = 1, 2, …, 21). Since we defined *k*_max_ = 3, the total KSAAP feature vector was thus 21 * 21 * 3 = 1,323.

### Amino acid composition (AAC)

AAC represents the frequency of each amino acid in a protein fragment. The fraction of 20 canonical amino acids can be calculated according to equation 1:

1 $${F}_{a}=\frac{{R}_{a}}{{\rm{R}}}$$

where R_a_ is the number of a given amino acid in a fragment and R denotes the length of the fragment^42^. The total AAC feature vector was 20.

### High quality indices (HQI)

High quality physicochemical indices were similar to those used to develop the hydroxylation site prediction tools, predHydroxy and RF-Hydroxysite^25,36^. Briefly, 8 indices describing various physicochemical properties corresponding to 8 groups were generated by grouping 544 associated amino acid properties in the AAIndex database using fuzzy clustering^43^. The 8 indices are (BLAM930101)^44^, (BIOV880101)^45^, (MAXF760101)^46^, (TSAJ990101)^47^, (NAKH920108)^48^, (CEDJ970104)^49^, (LIFS790101)^50^ and (MIYS990104)^51^. We extracted HQIs for each amino acid residue neighbouring the cysteine residue (regardless of whether it was modified or unmodified). The central residue was always C, which was not considered for window sites. Thus, the total HQI feature vector was 160.

### Oversampling to balance training data

Class imbalance, which happens when the sample sizes in the data classes are unequally dispersed^52^, has been identified as one of the most difficult issues in the machine-learning field^53^. Previous studies have described various approaches to address data imbalance^27–29^. A popular approach is random under-sampling, which decreases the number of sample points from the majority class such that the majority class is balanced with the minority class. However, the disadvantage of using this method is that it can dispose of conceivably valuable data that could be essential for classifiers^27^. Therefore, in this study, we utilized an alternative oversampling approach that randomly replicates data points from the minority class to balance with the majority class. The primary benefit of using this strategy is that there is no loss of information, as is seen when using an under-sampling approach^28,29^.

### Model learning and testing

To train our algorithm, we utilized a SVM-based machine learning strategy. SVMs are supervised machine learning algorithms used for many classification problems. SVM has been widely used in many bioinformatics problems, such as SUMOylation site prediction^54^ and protein fold recognition^55^. Briefly, SVM constructs a hyperplane in a high dimension space that separates two classes of attribute vectors using the largest distance margin. In this study, we used a radial basis function (RBF) kernel, which facilitates the classification of data that is not linearly separable^56^. This approach was initially performed on the training dataset using 2,203 features. The fidelity of the model parameters was assessed after each round of analysis based on 10-fold cross-validation and optimized in an iterative manner until no further improvement in model performance was observed. Two parameters, penalty (C = 0.1) and kernel width (kw = 0.005), were chosen based on the best predictive performance of our method.

To evaluate the performance of our method, we assessed four commonly used metrics: accuracy (ACC), sensitivity (SN), specificity (SP) and the Matthew’s correlation coefficient (MCC). These metrics, which have been used in previous studies to evaluate performance of other predictors^24,25^, are defined below:

2 $${\rm{ACC}}=\frac{{\rm{TP}}+{\rm{TN}}}{{\rm{TP}}+{\rm{TN}}+{\rm{FP}}+{\rm{FN}}}\times 100$$

3 $${\rm{SN}}=\frac{{\rm{TP}}}{{\rm{TP}}+{\rm{FN}}}\times 100$$

4 $${\rm{SP}}=\frac{{\rm{TN}}}{{\rm{TN}}+{\rm{FP}}}\times 100$$

5 $${\rm{MCC}}=\frac{({\rm{TP}})({\rm{TN}})-({\rm{FP}})({\rm{FN}})}{\surd ({\rm{TP}}+{\rm{FP}})({\rm{TP}}+{\rm{FN}})({\rm{TN}}+{\rm{FP}})({\rm{TN}}+{\rm{FN}})}$$

True positives (TP) indicate the number of correctly classified sulfenylation sites, true negatives (TN) represent the number of correctly classified negative sites, false positives (FP) denote the number of negative sites incorrectly classified as sulfenylation sites and false negatives (FN) indicate the number of actual sulfenylation sites incorrectly classified as negative sites. In addition, we also assessed the area under the receiver-operator characteristic (ROC) curve as an indicator of model performance. The ROC curve, which illustrates the trade-off between specificity and sensitivity^57^, yields a solitary execution measure called the area under curve (AUC) score, where the AUC for an arbitrary classifier is 0.5 and an AUC of 1.0 represents a perfect classifier^58^.

### Data availability

The data, the source code, and other materials for the work are available in the GitHub repository, @[https://github.com/HussamAlbarakati/SVM-Sulfosite].

## Electronic supplementary material

Supplementary Materials
