# Supplementary material for: SVM-SulfoSite: A support vector machine based predictor for sulfenylation sites
Source: Sci Rep. 2018 Jul 26;8:11288. doi: 10.1038/s41598-018-29126-x (PMC6062547; doi:10.1038/s41598-018-29126-x)
Supplement: Supplementary file 1 — Supplementary Materials [file 41598_2018_29126_MOESM1_ESM.docx]

**SVM-Sulfosite:A support vector machine based predictor for sulfenylation sites**

**Hussam J. AL-barakati**^1^**, Evan W. McConnell**^2^**, Leslie M. Hicks**^2^**, Leslie B. Poole**^3^**, Robert H. Newman**^4^ **& Dukka B. KC**^1,*^

1-Department of Computational Science and Engineering, North Carolina A&T State University, Greensboro NC 27411, USA. E-mail: dbkc@ncat.edu

2-Department of Chemistry, University of North Carolina at Chapel Hill, Chapel Hill, NC 27599.

3-Department of Biochemistry, Wake Forest University School of Medicine, Winston-Salem, NC 27157.

4-Department of Biology, North Carolina A&T State University, Greensboro NC 27411.

Supplementary Information File


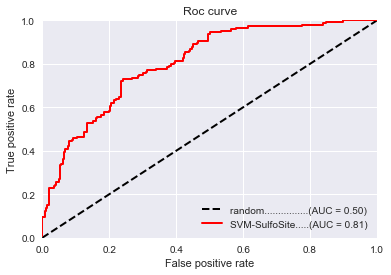


**Fig. S1.** Receiver operator characteristic (ROC) curve against the independent test set for Sulf-Rec (red).

**Table 5.** Shows 14 types of physicochemical properties with AAindex ID

| **NO** | **AAindex ID** | **Name of feature (References)** |
| --- | --- | --- |
| 1 | ARGP820101 | Hydrophobicity index (Argos et al., 1982) |
| 2 | NOZY710101 | Transfer energy, organic solvent/water (Nozaki-Tanford, 1971) |
| 3 | GRAR740102 | Polarity (Grantham, 1974) |
| 4 | CHAM820101 | Polarizability parameter (Charton-Charton, 1982) |
| 5 | CHOC760102 | Residue accessible surface area in folded protein (Chothia, 1976) |
| 6 | FASG760104 | pK-N (Fasman, 1976) |
| 7 | FASG760105 | pK-C (Fasman, 1976) |
| 8 | FASG760102 | Melting point (Fasman, 1976) |
| 9 | FASG760101 | Molecular weight (Fasman, 1976) |
| 10 | FASG760103 | Optical rotation (Fasman, 1976) |
| 11 | KLEP840101 | Net charge (Klein et al., 1984) |
| 12 | HUTJ700103 | Entropy of formation (Hutchens, 1970) |
| 13 | HUTJ700101 | Heat capacity (Hutchens, 1970) |
| 14 | HUTJ700102 | Absolute entropy (Hutchens, 1970) |

**References and definitions for the 14 physicochemical amino acid properties**

Hydrophobicity

AAindex ID: ARGP820101

AAindex name: Hydrophobicity index (Argos et al., 1982)

**Argos et al., 1982** (AAindex ref)**:**

Argos, Patrick, J. K. Rao, and Paul A. Hargrave. "Structural prediction of membrane‐bound proteins." *The FEBS Journal* 128.2‐3 (1982): 565-575.(PMID: 7151796)

Transfer energy, organic solvent/water

AAindex ID: NOZY710101

AAindex name: HTransfer energy, organic solvent/water (Nozaki-Tanford, 1971)

**Nozaki-Tanford, 1971** (AAindex ref)**:**

Nozaki, Yasuhiko, and Charles Tanford. "The solubility of amino acids and two glycine peptides in aqueous ethanol and dioxane solutions establishment of a hydrophobicity scale." *Journal of Biological Chemistry* 246.7 (1971): 2211-2217. (PMID: 5555568)

Polarity

AAindex ID: GRAR740102

AAindex name: Polarity (Grantham, 1974)

**Grantham, 1974** (AAindex ref)**:**

Grantham, R. "Amino acid difference formula to help explain protein evolution." *Science* 185.4154 (1974): 862-864. (PMID: 4843792)

Polarizability parameter

AAindex ID: CHAM820101

AAindex name: Polarizability parameter (Charton-Charton, 1982)

**Charton-Charton, 1982** (AAindex ref)**:**

Charton, Marvin, and Barbara I. Charton. "The structural dependence of amino acid hydrophobicity parameters." *Journal of theoretical biology* 99.4 (1982): 629-644. (PMID: 7183857)

Residue accessible surface area in folded protein

AAindex ID: CHOC760102

AAindex name: Residue accessible surface area in folded protein (Chothia, 1976)

**Chothia, 1976** (AAindex ref)**:**

Chothia, Cyrus. "The nature of the accessible and buried surfaces in proteins." *Journal of molecular biology* 105.1 (1976): 1-12. (PMID: 994183)

pK-N

AAindex ID: FASG760104

AAindex name: pK-N (Fasman, 1976)

**pK-N Fasman, 1976** (AAindex ref)**:**

Fasman, Gerald D. "Handbook of biochemistry and molecular biology. Lipids, carbohydrates, steroids-3." (1975).

“pK-N, it perhaps refers to the pK of the amino group either in the single free amino acid, or when it is at the N-terminus of a protein.”

pK-C

AAindex ID: FASG760105

AAindex name: pK-C (Fasman, 1976)

**pK-C Fasman, 1976** (AAindex ref)**:**

Fasman, Gerald D. "Handbook of biochemistry and molecular biology. Lipids, carbohydrates, steroids-3." (1975).

“pK-C, it perhaps refers to the carboxylate group at the C-terminus”

Molecular weight

AAindex ID: FASG760101

AAindex name: Molecular weight (Fasman, 1976)

**Molecular weight (Fasman, 1976)** (AAindex ref)**:**

Fasman, Gerald D. "Handbook of biochemistry and molecular biology. Lipids, carbohydrates, steroids-3." (1975).

Optical rotation

AAindex ID: FASG760103

AAindex name: Optical rotation (Fasman, 1976)

**Optical rotation (Fasman, 1976)** (AAindex ref)**:**

Fasman, Gerald D. "Handbook of biochemistry and molecular biology. Lipids, carbohydrates, steroids-3." (1975).

Net charge

AAindex ID: KLEP840101

AAindex name: Net charge (Klein et al., 1984)

**Net charge (Klein et al., 1984)** (AAindex ref)**:**

Klein, Petr, Minoru Kanehisa, and Charles DeLisi. "Prediction of protein function from sequence properties: Discriminant analysis of a data base." *Biochimica et Biophysica Acta (BBA)-Protein Structure and Molecular Enzymology* 787.3 (1984): 221-226. (PMID: 6547351)

Entropy of formation

AAindex ID: HUTJ700103

AAindex name: Entropy of formation (Hutchens, 1970)

**Entropy of formation (Hutchens, 1970)** (AAindex ref)**:**

Hutchens, John O. "Heat capacities, absolute entropies, and entropies of formation of amino acids and related compounds." *Handbook of biochemistry* (1970).

Heat capacity

AAindex ID: HUTJ700101

AAindex name: Heat capacity (Hutchens, 1970)

**Heat capacity (Hutchens, 1970)** (AAindex ref)**:**

Hutchens, John O. "Heat capacities, absolute entropies, and entropies of formation of amino acids and related compounds." *Handbook of biochemistry* (1970).

Absolute entropy

AAindex ID: HUTJ700102

AAindex name: Absolute entropy (Hutchens, 1970)

**Absolute entropy (Hutchens, 1970)** (AAindex ref)**:**

Hutchens, John O. "Heat capacities, absolute entropies, and entropies of formation of amino acids and related compounds." *Handbook of biochemistry* (1970).
